# Challenges to cross-sectoral care experienced by professionals working with patients living with low back pain: a qualitative interview study

**DOI:** 10.1186/s12913-020-4988-y

**Published:** 2020-03-04

**Authors:** Lisbeth Petersen, Regner Birkelund, Berit Schiøttz-Christensen

**Affiliations:** 1 Medical Research Unit, Spine Centre of Southern Denmark, Lillebaelt Hospital, Østre Hougvej 55, 5500 Middelfart, Denmark; 2 Health Services Research Unit, Lillebaelt Hospital, The Department of Regional health Research, University of Southern Denmark, Beriderbakken 4, 7100 Vejle, Denmark; 3 Medical Research Unit, Spine Centre of Southern Denmark, Lillebaelt Hospital, The Department of Regional health Research, University of Southern Denmark, Østre Hougvej 55, 5500 Middelfart, Denmark

**Keywords:** Cross-sectoral care, Continuity of care, Challenges, Professionals’ experiences, Course of treatment, Low back pain, Coherency, Qualitative study

## Abstract

**Background:**

While interdisciplinary, cross-sectoral collaboration promotes the effectiveness of rehabilitation programmes for persons with low back pain, challenges remain for this process. Few studies have explored challenges to cross-sectoral care as experienced by all the involved professionals across sectors during a course of treatment. The aim of this study was to explore challenges to cross-sectoral care as experienced by professionals involved in the course of treatment for patients with low back pain.

**Method:**

This semi-structured, qualitative interview study included 28 health care professionals and 8 social workers who interacted with patients with low back pain. A systematic text condensation method was used to analyse data. Nvivo was used to structure and thematise the interview data.

**Results:**

Professionals expressed challenges in relation to a lack of collaboration, knowledge sharing and acknowledgement of one other and they appeared to differ in their approach to patients with pain or patients with limited function. Additional challenges included time constraints, availability and subjective approaches to managing guidelines for low back pain. A lack of a common information technology (IT) registration system and limited knowledge of the work of other professions disrupted knowledge sharing among sectors.

**Discussion:**

The different approach to patients with pain or patients with limited function challenged mutual understanding and collaboration among professionals. The lack of mutual understanding and knowledge of each other’s work appeared to create an environment of disrespect and distrust among professionals that generated feelings of a lack of acknowledgement from other health care professionals.

**Conclusion:**

To provide cross-sectoral care, we must ensure that professionals work together towards transparent and informed transitions from one sector to the next. This study contributes to the existing literature by presenting challenges to cross-sectoral care that are experienced by the diverse groups of professionals involved in a course of treatment for patients with low back pain.

## Background

Cross-sectoral care encompasses referring and receiving aspects of the collaboration and is essential for persons with complex care needs [1]. Research demonstrated that interdisciplinary, cross-sectoral collaboration promotes rehabilitation through fewer and shorter hospitalisations, less postoperative complications, reduced dependency on help, a faster return to the labour market, reduced sick leave from work and higher patient satisfaction [2–4]. Despite these benefits, there are still barriers to cross-sectoral collaboration for persons with complex needs in Denmark and other countries [5–9]. Danish people who experience low back pain (LBP) represent a large group of patients that interacts with myriad health care professionals across sectors. These patients often end up circling the health care system with repeated assessments at primary or secondary health care facilities [10]. Prior to the current study, we explored LBP patients’ experiences of challenges during their course of treatment. Patients reported that they wanted acknowledgement of their situation living with LBP and more information about the background for referral. Patients experienced insufficient knowledge sharing, collaboration and support from professionals during their treatment (in review).

Cross-sectoral collaboration requires a high degree of coordination and communication between the primary care sector and municipalities [11], and national management guidelines have been formulated to support a common understanding in cross-sectoral collaboration [8, 9, 12]. These guidelines aim to ensure that patients with LBP receive the best possible treatment at the right time and that double treatment and overtreatment are avoided [13]. However, the experiences of patients with LBP have indicated that cross-sectoral care does not have the anticipated effect (in review).

Most back pain issues are handled in the primary care sector by general practitioners (GPs), chiropractors and physiotherapists. If a person experiences LBP, he or she can choose to consult his or her GP or a chiropractor. The GP will examine the patient, and if the GP does not find signs of serious pathology (red flags), the patient will be referred to physiotherapy (if needed). In Denmark, a consultation with a GP is covered by the National Health Service; with a referral from a GP, the price of consulting a physiotherapist is reduced by two thirds. A chiropractor performs the same examination as the GP, and if no red flags are identified, most patients are treated by the chiropractor in the clinic. Only one third of chiropractic care is covered by the National Health Service, and therefore it is quite expensive to the individual patient.

If the back pain has not improved after 8–12 weeks of treatment in primary healthcare, the patient is referred to a hospital or a Spine Centre for further diagnostics. Subsequently, there are several possible outcomes depending on the nature of the problem. One out of four patients is referred to rehabilitation at the municipal health service. In Denmark, a municipal is the smallest political and organisational unit; it serves to promote the comfort, safety, health and happiness of the citizens in the municipality. Municipal rehabilitation is a publicly paid service that extends over approximately 12 weeks in collaboration with physiotherapists employed by the municipality. Municipal rehabilitation often takes place in local rehabilitation centres.

As soon as a patient is registered with sick leave for more than 2 weeks, the local jobcentre will be involved. The jobcentres are owned by the Danish municipalities. These entities help unemployed citizens find jobs, and they are responsible for social benefits in the case of an illness that leads to disability. It is thus not uncommon for a patient to interact during the course of treatment with a GP, a physiotherapist, a chiropractor, a specialised Spine Centre, a municipal physiotherapist and a social worker from a jobcentre. The GP co-ordinates the treatment and is responsible for the overall health professional effort by regularly assessing the patient’s health. If needed, the GP contributes to the determination and continuous revision of treatment goals [13].

Few studies have explored challenges to cross-sectoral care as experienced by all involved professionals across sectors in a course of treatment [9, 14–17]. Most studies have considered one profession or established teams of multidisciplinary professionals [4, 18–21]. Others discussed cross-sectoral care within organisational or policy frameworks or the outcomes of collaboration [1, 22, 23]. The aim of the current study was to explore challenges to cross-sectoral care as experienced by professionals involved in the course of treatment for patients with LBP.

## Method

### Design

The project was conducted as a qualitative interview study. An inductive approach was used to allow patterns and themes to emerge from the empirical data before deciding on the theoretical approach [24]. Data were interpreted and analysed according to Malterud’s systematic text condensation strategy. The strategy represents a pragmatic approach to data analysis and comprises four steps: 1) total impression, from chaos to themes; 2) identifying and sorting meaning units, from themes to codes; 3) condensation, from code to meaning; 4) synthesising, from condensation to descriptions and concepts [25].

### Setting and participants

The study was anchored at a Danish Spine Centre, in collaboration with professionals from the primary and secondary sectors of five municipalities. The target group was health care professionals and social workers involved in the cross-sectoral course of treatment for patients with LBP.

Individual semi-structured interviews were conducted with 36 professionals who worked with patients with LBP, including 10 municipal physiotherapists, 8 social workers at the jobcentres, 8 GPs, 5 chiropractors and 5 physiotherapists from the primary care sector. All professionals were located in the area of the five collaborating municipalities.

The number of participants included in a qualitative interview study is typically around 15 ± 10. We estimated that 8–10 participants from each profession would be adequate in a study of this size. As expected, the last interviews with professionals from the same profession did not add any new knowledge to our study, a finding that suggested data saturation was achieved. The interviews were conducted from September 2016 to March 2017. They took place at the professional’s clinic or work space and lasted approximately 1 h.

GPs have busy schedules and are therefore often difficult to get involved in a study. With a mission to find a representative group of GPs, 30 were selected from a list of all GPs in the five municipalities based on location, size and number of referrals to the Spine Centre. The GPs were contacted by e-mail. Most declined to participate due to time constraints. Those who accepted came from clinics in both city and rural areas; they were either working alone or organised in a medical practice collaborative. The GPs were between 40 and 60 years old, and there were slightly more men than women.

Chiropractors and physiotherapists in the primary care sector see patients early in the course of treatment. We hypothesised that these professionals will be relatively independent of other sectors and experience fewer challenges in cross-sectoral collaboration. Thus, we interviewed only 5 in each group. To secure a representative selection of chiropractors and physiotherapists, we had help from 2 professionals working with cross-sectoral collaboration. This design resulted in 1 chiropractor and 1 physiotherapist from each of the five collaborating municipalities being interviewed, with equal numbers of men and women.

To secure a representative selection of participants from the municipalities, the physiotherapists and social workers were selected by their team leaders, who had the best knowledge of the degree to which professionals worked with patients with LBP. Ten physiotherapists and 10 social workers were invited to participate. One social worker declined and another got sick. The team leaders were unable to find replacements. The participants from the municipalities were between 25 and 55 years old, and two thirds were women. The division between women and men mirrors the general gender division among municipal physiotherapists and social workers. Social workers at the jobcentres stand out as separate from the other units in the health care system, partly due to a different set of legislation.

### Semi-structured interviews

The interviews used a semi-structured interview guide inspired by Kvale and Brinkmann [26] that contained an overview of the topics we wanted to explore and questions related to these topics. A semi-structured interview can be followed more or less stringently depending on the study and the interviewer. We wanted all our questions answered, but the order of questions was unimportant. The semi-structured interview also allows for new phenomena to emerge that the interviewer did not foresee [26]. Two researchers collaborated in developing the interview guide. The first researcher (the main author) is an anthropologist with 6 years of experience with interviews and participant observation. The second researcher is a consultant from the Region of Southern Denmark (see ‘Acknowledgements’) who has a Master of Social Science and more than 10 years of experience with interviews. The questions were piloted through the first two interviews within each profession and subsequently adjusted. Only minor adjustments were needed.

The interviews focused on the professionals’ experiences with patients with LBP. We asked about challenges they had experienced in relation to the patient’s course of treatment and about their collaboration with professionals from other sectors. The interview guide contained the following topics.

- The course of a consultation with patients having LBP:
  - Please describe a typical consultation with a patient having LBP. What kind of information is important for you to get from the patient?
- The professional’s view on his or her role and responsibility in relation to the patient’s course of treatment:
  - How do you perceive your role in the patient’s course of treatment?
- Collaboration with other professionals across sectors:
  - With which other professionals do you collaborate?
  - How do you collaborate with the different professionals?
  - What information is important for you to get from other professionals with whom the patient interacts?
- The professional’s perception of challenges in relation to the patient’s course of treatment or his or her practice:
  - In which situations do you find that a course of treatment is coherent?
  - In which situations do you find that the course of treatment is challenged?
  - From your perspective, what are the biggest challenges to cross-sectoral care?
- Suggestions for improvements
- Explanatory models for cause and treatment of pain: which information is given to the patients?
  - What information is important for you to tell your patients?

There were small variations in the questions depending on which group of professionals were interviewed.

The interviews were conducted by the two researchers who developed the interview guide. One led the interview and ensured that all topics in the interview guide were discussed. The other observed and listened to the interview while taking additional notes. This set-up gave the observer time to reflect on statements from the participant and ask in-depth questions that the interviewer might have missed while focusing on the flow of the interview.

### Ethics

According to Danish law, ethical approval from the National Committee for Health Research Ethics is not required for conducting interviews as long as the data are used anonymously. The project was registered with the Danish Data Protection Agency number 18/27313, and permission was granted by the Spine Centre management. Those interviewed were informed through oral and written information.

### Data analysis

The interviews were recorded and transcribed verbatim; the programme Nvivo was used to structure and thematise the interview data. The data were interpreted and analysed according to Malterud’s systematic text condensation strategy [25], which gave an overview and helped structure the interview data. Four researchers collaborated in the meaning-condensation and analysis phases: the 2 interviewers presented in the ‘Semi-structured interviews’ section and the authors of this manuscript. The second author is a professor of patient-centred cancer treatment with comprehensive experience in the qualitative research area. The third author is a rheumatologist with more than 20 years of experience as an interdisciplinary team leader. The analytic process focused on the meanings, patterns and important characteristics of professionals’ experiences on the course of treatment for patients with LBP [25]. The interview data were divided into themes from the patterns generated by re-reading the data, and the essential themes were then linked together in descriptive statements. Thus, the method involved condensation of expressed meanings of increasing significance regarding professionals’ experiences of cross-sectoral care for patients with LBP. The themes were subsequently presented and verified by the participants as well as other health care professionals through five workshops where the themes were discussed.

## Results

The interview data revealed differences among the professional groups and their approaches to dealing with patients with LBP. There were also differences within the professional groups. Myriad challenges to a coherent course of treatment were thus articulated. However, three main themes emerged from the interview data and are presented in the following paragraphs:

1. Lack of collaboration;
2. Lack of knowledge sharing;
3. Lack of acknowledgement.

### Lack of collaboration

Professionals in different sectors generally expressed a belief that cross-disciplinary collaboration promoted coherency and improved treatment for patients with LBP. Professionals spoke about collaboration as an active form of communication where professionals with different professions from different sectors discuss a patient’s situation to achieve the best possible treatment. The professionals did not share a mutual understanding and a common goal for the course of treatment.

The physiotherapists believed their role was to help patients increase their functional capacity and to educate them on how to manage their back pain in everyday life. Their focus was on function and what the patient can do to live a valuable everyday life despite back pain. The social workers followed a different set of legislation, and their goal for the patients was to keep them in their jobs or to get them back to employment as soon as possible. To accomplish these tasks, they required a diagnosis and the guidelines that follow this diagnosis.

Among the professions the goal for the patient’s course of treatment appeared to differ. This finding was illustrated by the following statements from professionals in the municipal services, when asked what would be the best approach for the course of treatment for patients with LBP:

"Give them time and peace to process the situation. Then they will find a way out of it, I’m sure. People are good at finding solutions to problems, but in this system, it is hard for them to find peace for rehabilitation” (municipal physiotherapist 10).

“The best course would be the fastest possible way through diagnosis and treatment. What needs to be done in order to get clarification?” (municipal social worker 4).

Despite they agreed that collaboration promoted coherence, practice appeared to be more problematic. The municipal physiotherapists felt that collaboration between professionals in the municipalities was difficult due to different mindsets:

“Interdisciplinary collaboration between social workers and us as physiotherapists is very challenging because we really do not cooperate very much. We try, but it's really difficult. We have very different approaches to our patients on what is best for them and where they should go. It’s just two very different cultures and mind-sets” (municipal physiotherapist 9).

One example of a mismatch between professionals that challenged collaboration and made it difficult to sustain coherency was the contradiction of the distinction between function and diagnosis.

Professionals from all sectors noted that limited communication due to lack of availability prevented collaboration. Most of those interviewed had experienced that professionals in other sectors were difficult to reach. Contacting a professional by phone was time-consuming, and many participants said that they sometimes felt that they were disturbing the person when they finally reached him or her:

"Well, sometimes it could be easy if you could just call a GP and get a short dialogue about what does this information mean, or what are your thoughts about this patient, how much are we allowed to push? We often have limited time to make the phone call” (social worker 7).

The professionals cited busy schedules as the reason for not making the call, and most communication was therefore through e-mail correspondence, Messenger and other digital communication tools. Most professionals thought this kind of communication was the best option, but the communication between sectors was also described as casual. This factor limited the possibilities for professionals to share knowledge, discuss patients’ problems and ensure an informed and visual treatment plan based on interdisciplinary consensus.

### Lack of knowledge sharing

The professionals used referrals, health records or a rehabilitation plan as tools to share knowledge about the patient from one clinician to the next. Almost every professional expressed a wish for a common information technology (IT) registration system that allowed the patient’s health record to be shared by professionals across sectors:

“I would like a more direct line of knowledge sharing between me and the next clinician because we have the impression that the knowledge we generate and pass on to the GP gets lost in transfer. Even though we have conducted a thorough clinical examination and a lot of useful information, it does not transfer any further. You cannot look into each other's systems, and therefore the patients are messengers and that’s not good enough” (physiotherapist from the primary care sector 2).

The professionals expressed that transition of knowledge from one sector to another served to minimise repetition by building on knowledge that was already generated. One challenge for a coherent treatment course was the lack of a common IT system where knowledge of the patient could be shared by all professionals working with the patient. The professionals expressed that knowledge sharing provides the opportunity for a mutual understanding of each other’s work, policies, procedures and work cultures. Most of the social workers believed that successful knowledge sharing requires a distinct division of roles with agreement on who was responsible for what during the course of treatment:

“We need the doctor’s objective professional opinion, that’s really important. Sometimes we have seen from the Spine Centre some recommendations of how many hours the patients can go to work on a weekly basis, but it’s not always possible for us to meet these recommendations due to legislation. I think that recommendations like that are not supposed to be discussed at the Spine Centre, that’s our job at the municipal jobcentre” (social worker 3).

The social workers argued that by staying within their own remit, professionals could give the correct information to patients and avoid working against other professionals by creating expectations that could not be met. The interview data showed that the professionals were often insecure or misinformed about what the patient would face when referred to another sector. This fact was evident when we asked the professionals what they did with patients and what they told them to expect with a referral. The information on what to expect did not always match the care that patients actually received.

During the interviews with social workers, it became clear that they faced challenges due to the lack of knowledge and consensus on expectations about treatment goals. They expressed that they knew little about the nature of back pain and were often in doubt when dealing with patients with LBP.

"The patient follows a rehabilitation plan but after the rehabilitation is completed, what then? I really would like to know that. Is it just, ‘bang’ back to work? Or is it normal that you experience back pain afterwards? Because maybe it is normal for a patient to still have some back pain after rehabilitation is complete, but I don’t know, and the patient doesn’t know either. I could use some information about what to expect with this patient. Where should I begin, and which specific needs should I pay attention to?” (social worker 6).

The social worker expressed doubt on how to act because of lack of a common understanding of LBP and the procedure and expectations for the patient. The social workers rarely succeeded in calling a health care professional to clarify these questions, and thus they were left with only one option: refer the patient back to the GP, which, according to several professionals, would start a new wave of assessments in the primary health care sector.

### Lack of acknowledgement

The health care professionals agreed that there should be progress from one health facility to the next, but they also said that it is important for them to perform certain assessments ensure quality care.

“We always start from scratch. Analysis of posture and movement pattern and we talk about their back pain and so on. It would be dangerous just to rely on the information in the GP’s referral and just work with that because there could be other relevant things as well” (physiotherapist from the primary care sector 4).

“We do, of course, examine the patient ourselves. Maybe the previous professionals also did the examination, but just to be sure that the therapist or the doctor has done a thorough assessment and has been ‘all around the patient” (municipal physiotherapist 2).

During the interviews, there were many examples of professionals talking about each other with mistrust.

GPs said that they primarily referred their patients to physiotherapy because of tradition and current referral guidelines, while chiropractors felt that this action reflected a lack of confidence in their skills. Several chiropractors suggested that that GPs should refer more patients to chiropractors because they perform the same assessment as physiotherapists but have shorter waiting lists. Chiropractors generally expressed frustration that their competencies were underestimated. Some chiropractors experienced that GPs did not listen to them, and in some cases the chiropractors felt there were in opposition with the GP. Indeed, the GP could withdraw the treatment plan that the chiropractor put into operation. The physiotherapists, on the other hand, were more dependent on the GP’s referral and expressed a wish for more feedback from the GP. The physiotherapists did not see value in working with the chiropractors because physiotherapists often treated the patient themselves, whereas chiropractors found it valuable to combine chiropractic management with physiotherapy.

The interview data showed that most of the challenges to collaboration and a coherent course of treatment were articulated by professionals in the municipalities. Social workers expressed that they often felt opposed by the health care professionals or that the health care professionals had a tendency of taking the patient’s side, leaving the social worker feeling that they stood alone against both patient and health care system:

“Sometimes there are suddenly a lot of people around the citizen that stand against us and the plan we would like to have for the citizen, and that’s where our work gets really, really difficult” (social worker 1).

Municipal physiotherapists said that they sometimes felt that their knowledge of the patient’s current situation and progress was neglected when social workers contacted the GP to get a report on a patient’s health status. The physiotherapist argued that they knew the patient’s situation better than the GP and could therefore give a detailed report on the patient’s current health status.

The feeling of that other professionals did not acknowledge others’ efforts appeared to create an environment of negativity or distrustfulness that challenged cross-sectoral care because it hindered professional’s inclination to collaborate.

## Discussion

This interview study showed that professionals experience challenges in relation to collaboration, knowledge sharing and acknowledgement of each other. In the following section, we discuss our results in comparison to the literature and discuss how our findings affect patients’ experiences in their course of treatment for LBP, as shown in a former study (in review).

### Lack of collaboration

According to our findings and existing literature, collaboration can be challenged by lack of time, accessibility and knowledge sharing among sectors [8, 14, 27]. Poitras et al. evaluated barriers to the use of management recommendations aimed at preventing LBP disability with general practitioners, physiotherapists and occupational therapists working in Quebec, Canada [9] and the use of LBP guidelines by occupational therapists [8]. They found that the GPs struggle with short treatment sessions, limited frequency and long intervals in follow-up sessions [9]. Similarly, in an evaluation of the use of a LBP management protocol in the Capital Region of Denmark, Buch et al. showed that GPs, physiotherapists and chiropractors do not follow the guidelines primarily due to time pressure [27]. Thus, follow-up practice for individual cases may be influenced by time constraints.

The Danish Health Authority initiated the development of guidelines for cross-sectoral courses of treatment for patients with LBP [11]. However, these procedures were not implemented as specific steps that detailed collaborations; rather, they serve as guidelines that can be interpreted in different ways by local health care professionals. A lack of formal collaboration structures, combined with time constraints and limited availability of other professionals, may cause professionals to practice their normal routines instead of following the LBP guidelines as suggested by The Danish Health Authority. Similar to our study, Poitras et al. identified the barriers to cross-sectoral collaboration as a lack of a common goal, time, availability of other professionals and formal collaboration structures [8].

### Lack of knowledge sharing

We found that lack of knowledge sharing inhibited cross-sectoral care because professionals lacked consensus and understanding of the expectations and work of professionals in other sectors. Additionally, knowledge was often not transferred from one sector to the next. Poitras et al. showed that occupational therapists, whose role can be compared to that of physiotherapists in a Danish context, often experience delays or under-referrals due to the lack of knowledge about the contribution of occupational therapists to the management of LBP disability [9]. Buch et al. reported that the development of knowledge and interdisciplinary relationships, which should contribute to more comprehensive referrals, has been given less priority [27]. Thus, the relationships among professionals across sectors and disciplines appears to be important for knowledge sharing to succeed. The development of interdisciplinary relationships requires mutual respect and trust in each other’s knowledge and skills. However, our results suggest these areas are currently lacking.

### Lack of acknowledgement

Our results revealed an interesting paradox. Although professionals experienced that the information they obtained from the patient was neglected in the transmission from one sector to another, they themselves did not use the information that was passed on to them when they started their conversation with the patient. Professionals therefore seem to reproduce the very same practice that they find dissatisfactory.

The expressed lack of recognition of each other’s work, lack of knowledge sharing and an emphasis on the professionals’ own core output suggest that professionals have their own agenda for the patient’s course of treatment that is reflected in their collaboration and relationships with other professionals (who may also have a different agenda). The professional’s role during the course of treatment is apparently considered more important than a common goal for and with the patients. This view, in turn, contributes to undervaluing the work of collaborators.

Several reasons may underlie this devaluation. It is not always easy to make an exact diagnosis for patients with LBP. Back pain cannot always be explained from a biomedical perspective, and the health care professional may need to work from a biopsychosocial approach, namely by incorporating knowledge of several aspects of the patient’s daily life to the assessment of the back pain situation [6, 28]. Buch et al. showed that implementation of LBP guidelines in hospitals and municipalities focuses on the professional core output [27]. Moeller explored health professionalism in Danish municipal rehabilitation centres and found that the core output differs greatly among health care professionals, depending on whether they work within a biomedical or biopsychosocial paradigm [28]. This discrepancy may explain why professionals do not immediately incorporate knowledge generated by others who may differ in their approach to pain and function. They instead prefer to examine the patient themselves and gain their own understanding of the patient’s experience with LBP. Health care professionals must keep medical records, which mean they have to record their own findings and thus possibly repeat the patient assessment. Studies on the challenges to cross-sectoral care that include professionals who work with disorders other than LBP have revealed similar challenges. These trials include poor collaboration due to lack of knowledge about duties and responsibilities of other professionals involved in the care process, lack of a common IT registration system, limited knowledge sharing and conflicting perceptions of each other’s roles and images [14–17].

### Influencing patient experiences

We previously explored patients’ experiences of their course of treatment for LBP (in review). Adding the statements from professionals provides an understanding of the context in which the perceived challenges for cross-sectoral care are embedded. In the study of LBP patients’ experiences with their course of treatment, we showed how lack of collaboration towards a common goal challenges a coherent course of treatment and inspires patients to feel frustrated and sometimes trapped between distinct agendas from different professionals. Moreover, we argued that patients experience a lack of acknowledgement and called for a person-centred approach that includes a biopsychosocial view on LBP and a focus on the patients’ own goals and preferences (in review). Comparing this finding to our current analysis, some professionals appear to focus on their individual professional core output rather than the patient’s experiences of living with LBP. This emphasis leaves the patients feeling that they are not being heard and their needs are neglected.

Moeller’s study showed that—parallel to a biopsychosocial approach—municipal professionals also use a medical discourse to oppose the dominant medical group, e.g., by underlining other professionals’ lack of human perspective. This view enhances the rehabilitation centres as a constructive and forward-looking alternative [28]. Neglecting and/or talking negatively about the work performed by former professionals are examples of such a discourse. However, according to our study on patients’ experiences, such a strategy can have the opposite effect. Our patient study showed how negative stories about other professionals are perceived by patients as an expression of poor collaboration and rivalry among professionals. These accounts made the patients feel uncomfortable and distrustful towards the professionals (in review). Opposing discourses can also result in different explanatory models provided by professionals. Our study on patients’ experiences showed how they often experience receiving contradictory information from different professionals across sectors. This phenomenon challenges the patients’ perception of coherence and understanding of their course of treatment (in review).

### Strengths and limitations

Semi-structured interviews provided valuable material on how professionals perceive and experience the course of treatment for patients with LBP.

Collecting data on patients’ and professionals’ experiences of cross-sectoral care allowed us to compare statements and approaches between patients and professionals and among professionals. This comparison revealed several discrepancies between the patients’ needs and the professionals’ practice that we would not have seen if we had talked to professionals or patients alone.

Given our (the researchers) distinct educational backgrounds, experience and knowledge of the field, we had very different approaches to data analysis, which influenced the discussions in the interpretation of data. This method supported the academic breadth of the analysis.

Interviewing professionals from both primary health care and municipalities offered valuable information on the experiences of those involved in treatment. However, the professionals worked in very different contexts, and so it was challenging to compare experiences across sectors. Organizational, cultural and political circumstances may have influenced our analysis without our awareness.

We only interviewed 5 chiropractors and 5 physiotherapists from primary care, but the interviews emphasised related topics and the information was similar. Thus, the number of interviews was adequate for data saturation. To ensure a representative selection of chiropractors and physiotherapists, 2 professionals who work with cross-sectoral collaboration were responsible for selecting and making agreements with chiropractors and physiotherapists. It is possible, however, that these participants were selected due their approach to patients with LBP or were already known to the selectors rather than randomly chosen, leading to misclassification of important information.

During the interviews, the researchers experienced both positive and negative attitudes towards other professional groups, to patients with LBP, to LBP guidelines and collaboration and the researchers who represented the Spine Centre. Thus, we cannot be sure that these participants were representative, but we believe that the barriers described in this material cover most of the barriers that occur. Furthermore, we believe that the material will form the basis for future interventions in order to strengthen collaboration across sectors.

### Implication for practice

Our results showed that interdisciplinary knowledge sharing is crucial if health care professionals are to provide coherency in cross-sectoral care. There are several methods to achieve this goal. For example, professionals can establish interdisciplinary networks, as suggested by Gibbs et al. [29], where all involved professionals can meet either in a physical or virtual space to discuss a specific case and share knowledge.

During the interviews, several professionals called for a key worker to coordinate care, a view also described by Morgan et al. [30]. We consider the use of a key worker to support patients with LBP useful, and based on our current knowledge, we suggest that it could be a health care professional from primary health care working closely with the GP.

In order to support communication and knowledge sharing among professionals, it may be beneficial to have a common e-portal where relevant health care professionals have access to the patient’s careplan. Although this idea was expressed by several professionals during this study, it is not an easy task to develop due to rules on data protection and protection of personal data/rights. However, during this study we began this process by developing a set of cards with descriptions of specific LBP diagnoses, legislation, roles of the different groups of professionals and other relevant data that can be given to the patients to carry with them during their course of treatment. The idea is that these cards will eventually be converted to an e-portal.

## Conclusion

This qualitative study demonstrated that cross-sectoral care is challenged by a lack of collaboration, knowledge sharing and acknowledgement between professionals across sectors. Professionals who are involved in the course of treatment for patients with LBP differed in their approach to pain and function. These discrepancies challenged mutual understanding and collaboration among professionals. Further challenges included time constraints, availability and subjective approaches to manage LBP guidelines. The dearth of a common IT registration system and limited knowledge of the work of other professions restricted the knowledge sharing between sectors. The lack of mutual understanding and knowledge of each other appeared to create an environment of disrespect and distrust among professionals that promoted feelings that other health care professionals did not acknowledge one another’s work.

This study contributes to the existing literature by presenting challenges to cross-sectoral care as experienced by the distinct groups of professionals involved in the course of treatment for patients with LBP. Together with our article on LBP patients’ experiences during their course of treatment, this report offers a unique analysis of professionals’ experiences of cross-sectoral care and their views on personal, individual, organisational and cultural challenges to cross-sectoral care.

If health care professionals are to provide adequate and effective cross-sectoral care, they must ensure that they work together towards transparent and informed transitions from one sector to the next. This goal includes working towards a common goal. Based on our current knowledge, we suggest that future studies should focus on the different cultures, attitudes and understanding among health care professionals in order to establish an environment based on trusting collaboratives between professionals working with cross-sectoral care.

## Data Availability

The datasets generated and/or analysed during the current study are available from the corresponding author upon reasonable request.

## Abbreviations

GP: General practitioner
LBP: Low back pain
